# Outcomes of endoscopic submucosal dissection for gastroesophageal reflux disease (ESD-G) for medication-refractory gastroesophageal reflux disease: 35 cases underwent ESD-G including 15 cases followed more than 5 years

**DOI:** 10.1186/s12876-021-02022-x

**Published:** 2021-11-18

**Authors:** Kazuhiro Ota, Toshihisa Takeuchi, Yuichi Kojima, Noriaki Sugawara, Shinya Nishida, Taro Iwatsubo, Shimpei Kawaguchi, Satoshi Harada, Satoshi Tokioka, Kazuhide Higuchi

**Affiliations:** 1 Second Department of Internal Medicine, Osaka Medical and Pharmaceutical University, 2-7 Daigaku-machi, Takatsuki, Osaka 569-8686 Japan; 2 Department of Internal Medicine, Katsuragi Hospital, Kishiwada, Osaka Japan; 3 Department of Gastroenterology, First Towakai Hospital, Takatsuki, Osaka Japan

**Keywords:** Endoscopic surgery, Proton pump inhibitor, Gastroesohageal reflux disease, ESD-G

## Abstract

**Background:**

Although some kinds of endoluminal surgery for patients with proton pump inhibitor (PPI)-refractory gastroesophageal reflux disease (GERD) have been reported, there are few reports on their long-term outcomes. In 2014, we reported the effectiveness of endoscopic surgery for PPI-refractory GERD, which we invented and named endoscopic submucosal dissection for GERD (ESD-G) in 2008. Thereafter, we accumulated more cases and monitored the patients’ condition postoperatively and describe the outcomes herein.

**Patients and methods:**

This single-center, single-arm trial was conducted at the Osaka Medical and Pharmaceutical University Hospital. We compared outcomes between before and 3–6 months after ESD-G. Additionally, we investigated the outcomes of patients 5 or more years after ESD-G.

**Results:**

We performed 42 ESD-G procedures in 35 patients between 2008 and 2020. In seven patients, ESD-G was performed twice for various reasons. The frequency scale for the symptoms of GERD score was significantly improved 3–6 months after ESD-G (22 → 10, *p* < 0.0001); the Los Angeles classification for reflux esophagitis was clearly improved after ESD-G (*p* = 0.0423). The number of reflux episodes was not decreased by ESD-G. There was a significant difference in the potency unit of gastric acid secretion suppressants for controlling GERD-related symptoms between baseline and 3–6 months after ESD-G (*p* = 0.0009). In patients without a history of distal gastrectomy who underwent ESD-G, the potency unit of gastric acid secretion suppressants significantly decreased 5 or more years after ESD-G (*p* = 0.0121).

**Conclusion:**

ESD-G may be effective in patients with refractory GERD-related symptoms without a history of distal gastrectomy.

## Introduction

Gastroesophageal reflux disease (GERD) is divided into reflux esophagitis with esophageal mucosal injury and non-erosive reflux disease (NERD) without mucosal injury. Although some patients with esophageal mucosal injury may be asymptomatic, patients with NERD may have severe symptoms without esophageal mucosal injury. In addition, the degree of mucosal injury does not necessarily correlate with symptoms [1–4]. NERD accounts for more than half of all GERD cases, is more common in women and non-obese individuals, is less commonly associated with hiatal hernia, is less likely to respond to medical treatment, and is considered a separate entity rather than a mild form of reflux esophagitis. Therefore, treatment methods should be tailored to each condition.

Since the speed of healing and resolution of GERD symptoms in patients with reflux esophagitis depends on the acid-secretory inhibitory properties of drugs, the use of potent acid-secretory inhibitors is recommended for treatment [5]. It is important to administer rapid, continuous, and potent acid suppression for 24 h at an early stage, followed by a minimum level of acid suppression in consideration of symptoms and recurrence of esophageal mucosal injury [6, 7]. However, high-dose proton pump inhibitors (PPIs) have not been shown to improve GERD symptoms in patients with NERD who do not respond to acid-secretory medications [8, 9]. These patients are considered to have reflux of substances other than acid, and high-dose PPIs are considered ineffective because no amount of acid secretion suppression is effective. When acid secretion control therapy alone is not effective, mosapride [10] and acotiamide [11], which improve gastrointestinal peristalsis, and Chinese herbs [12, 13] have been reported to be effective.

Most patients with GERD can achieve mucosal healing and symptomatic improvement with medical therapy, but some patients do not respond to medical therapy alone. Such cases are referred to as PPI-refractory GERD. It is also possible that even when medical therapy is effective, GERD will flare up due to dose reduction or discontinuation, resulting in a permanent need for medication. Surgical treatment has also been proposed for PPI-refractory and dependent GERD. The current standard of care, laparoscopic gastroesophageal reflux prevention, has been widely compared to long-term PPIs in terms of adherence, cost, safety, and efficacy, but the pros and cons of aggressive surgical treatment are controversial because of the risks involved [14, 15]. Although some kinds of endoluminal surgery for patients with PPI-refractory GERD have been reported previously, there are few reports on its long-term outcomes [16–24]. We have reported the effectiveness of endoscopic surgery for PPI-refractory GERD, named endoscopic submucosal dissection for GERD (ESD-G) in 2014 [19], and this procedure was described in the latest GERD guideline published by the Japanese Society of Gastroenterology in 2021 [25]. Since this report, we continued to accumulate more cases and monitor the patients’ condition after this procedure. We herein describe the short-term outcomes of additional cases and outcomes of cases with long-term follow-up.

## Patients and methods

### Study design

The present study was a single-center, single-arm trial conducted at the Osaka Medical and Pharmaceutical University Hospital. The study was conducted in accordance with the 1975 Declaration of Helsinki (as revised in 1983). The protocol was approved by the ethics review committee at Osaka Medical and Pharmaceutical University (No. 0563, May 12, 2008 and No. 1507, March 3, 2016) and registered in the University Hospital Medical Information Network Clinical Trial Registry (UMIN000042538).

### Procedure and study population

**Fig. 1:**
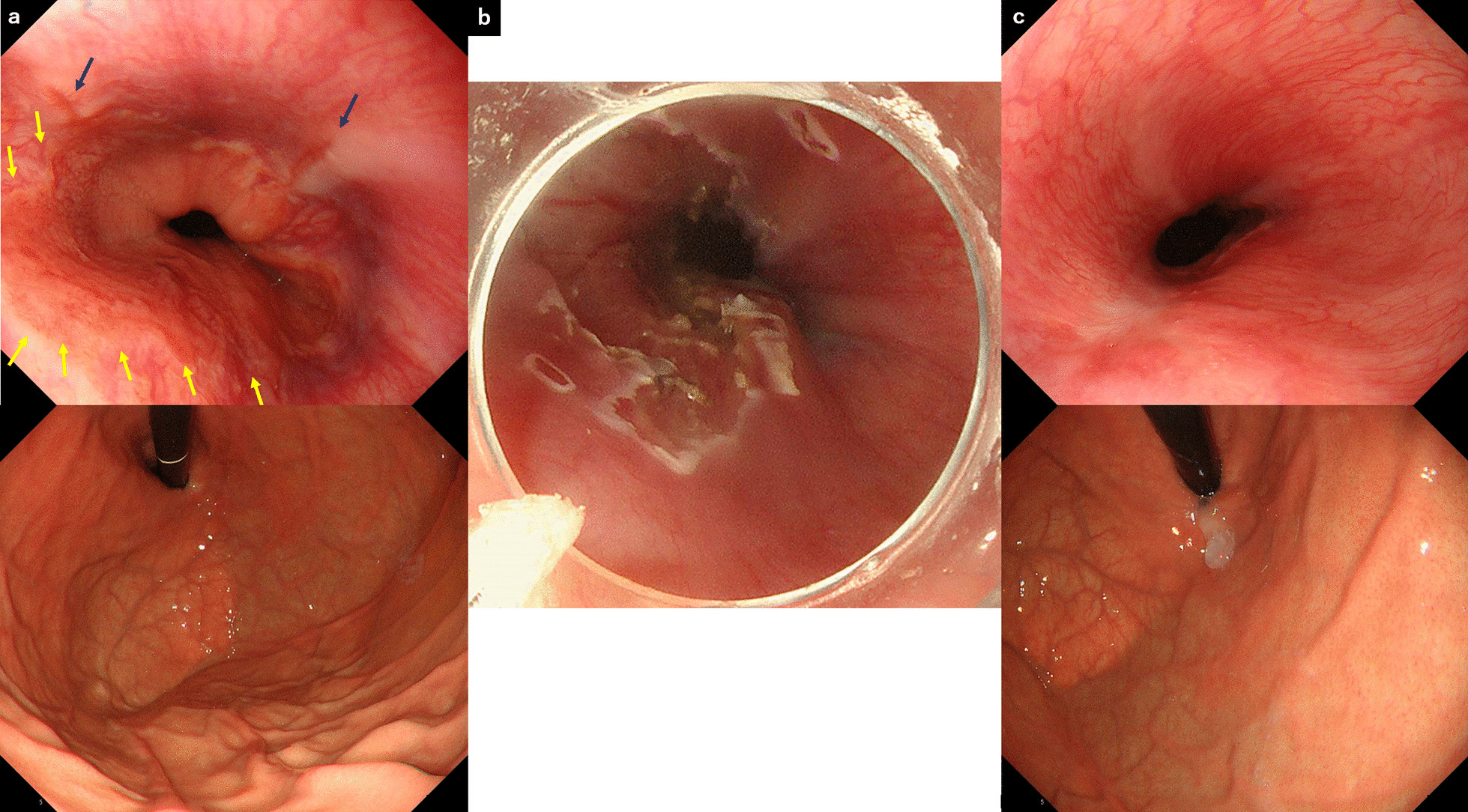
Endoscopic findings of the esophagogastric junction in a patient who underwent endoscopic submucosal dissection for gastroesophageal reflux disease (ESD-G). Before ESD-G (**a**), immediately after ESD-G (**b**), and 3 months after ESD-G (**c**). The area enclosed by the yellow arrows is the Barrett epithelium, and the mucosa of the same area is resected. Dark blue arrows indicate reflux esophagitis, and reflux esophagitis has disappeared after ESD-G

The main purpose of ESD-G is to narrow the hiatal opening by performing mucosal resection of the esophagogastric junction (EGJ) mucosa using endoscopic submucosal dissection (ESD) [19]. The resultant scarring narrowed the lumen of the hiatal opening, suppressing gastric reflux. The resection was limited to half (or one-quarter + one-quarter) of the EGJ lumen circumference. Previous reports indicated that the risk of stricture development increases following esophageal ESD involving more than three-quarters of the esophageal lumen circumference [26]. The semi-peripheral mucosa around the lesion site where Barrett’s epithelium or reflux esophagitis was present was resected. The resection area of the oral and anal sides were the upper end of Barrett’s epithelium and lower end of the esophageal hiatal hernia, respectively (Fig. 1a–c). ESD-G was performed by endoscopists with sufficient experience performing esophageal ESD (KO, SK, SH, YK, TT, and ST). The inclusion criteria of ESD-G were patients with endoscopic findings suggesting the presence of GERD such as reflux esophagitis, cloudiness of the lower esophageal mucosa, or presence of Barrett’s epithelium and with GERD-related symptoms that persisted despite PPI therapy for 8 weeks. High-resolution esophageal manometry was used to exclude patients with possible esophageal functional diseases other than GERD. Therefore, esophageal achalasia and its analogous diseases had been excluded from this study. Each subject was 20–65 years old at the time that written consent was obtained; each participant received oral and written explanations of the study.

### Evaluations

We compared the following items between before and 3–6 months after ESD-G in patients who underwent ESD-G: frequency scale for the symptoms of GERD (FSSG) [27], Los Angeles classification of the endoscopic findings, number of reflux episodes during a 24-h esophageal multichannel intraluminal impedance (MII) monitoring study, and the potency unit of gastric acid secretion suppressants for controlling GERD-related symptoms. Regarding the potency unit of gastric acid secretion suppressants for controlling GERD-related symptoms, we also evaluated patients 5 years after the procedure.

We investigated the following outcomes of all patients 5 or more years after ESD-G: the current potency unit of gastric acid secretion suppressants for controlling GERD-related symptoms, additional surgical procedures, and the difference in symptoms from before ESD-G.

In the present study, the potency unit of gastric acid secretion suppressants for controlling GERD-related symptoms was defined as “1” and equaled by 20 mg of omeprazole, 30 mg of lansoprazole, 10 mg of rabeprazole, and 20 mg of esomeprazole. The potency unit of vonoprazan (20 mg) was defined as “4” in reference to our previous report [28].

### Twenty-four-hour esophageal multichannel intraluminal impedance (MII) monitoring study

The catheter for pH and impedance monitoring was inserted with X-ray guidance, and the esophageal pH sensor was positioned at the distal end of the esophagus, 5 cm from the point of the crossing diaphragm as the EGJ. PPI treatment should have been ceased prior to 24-h pH and impedance monitoring; however, there were many cases wherein PPI treatment could not be discontinued; therefore, impedance alone was evaluated in this study. For esophageal MII monitoring, the number of refluxes was evaluated. In our study protocol, the diagnosis results, such as reflux esophagitis, true NERD, reflux hypersensitivity, or functional heartburn, were not included in the analysis.

### Statistical analysis

Differences in the means of continuous data were compared using the paired *t* test and Wilcoxon signed-rank test. Differences in the means of categorical variables were compared using the Fisher exact test. A *p* value < 0.05 was considered significant; all tests were two-sided. All statistical analyses were performed using JMP Pro version 15 software (SAS Institute Inc., Cary, NC, USA).

## Results

We performed 42 ESD-G procedures in 35 patients between 2008 and 2020. The mean time for the ESD-G procedure was 52.0 (standard deviation [SD]: 28.6) min. Complications were observed in four cases: three patients with stenosis underwent endoscopic balloon dilation and one patient with bleeding underwent endoscopic hemostasis.

### First endoscopic submucosal dissection for gastroesophageal reflux disease

**Fig. 2:**
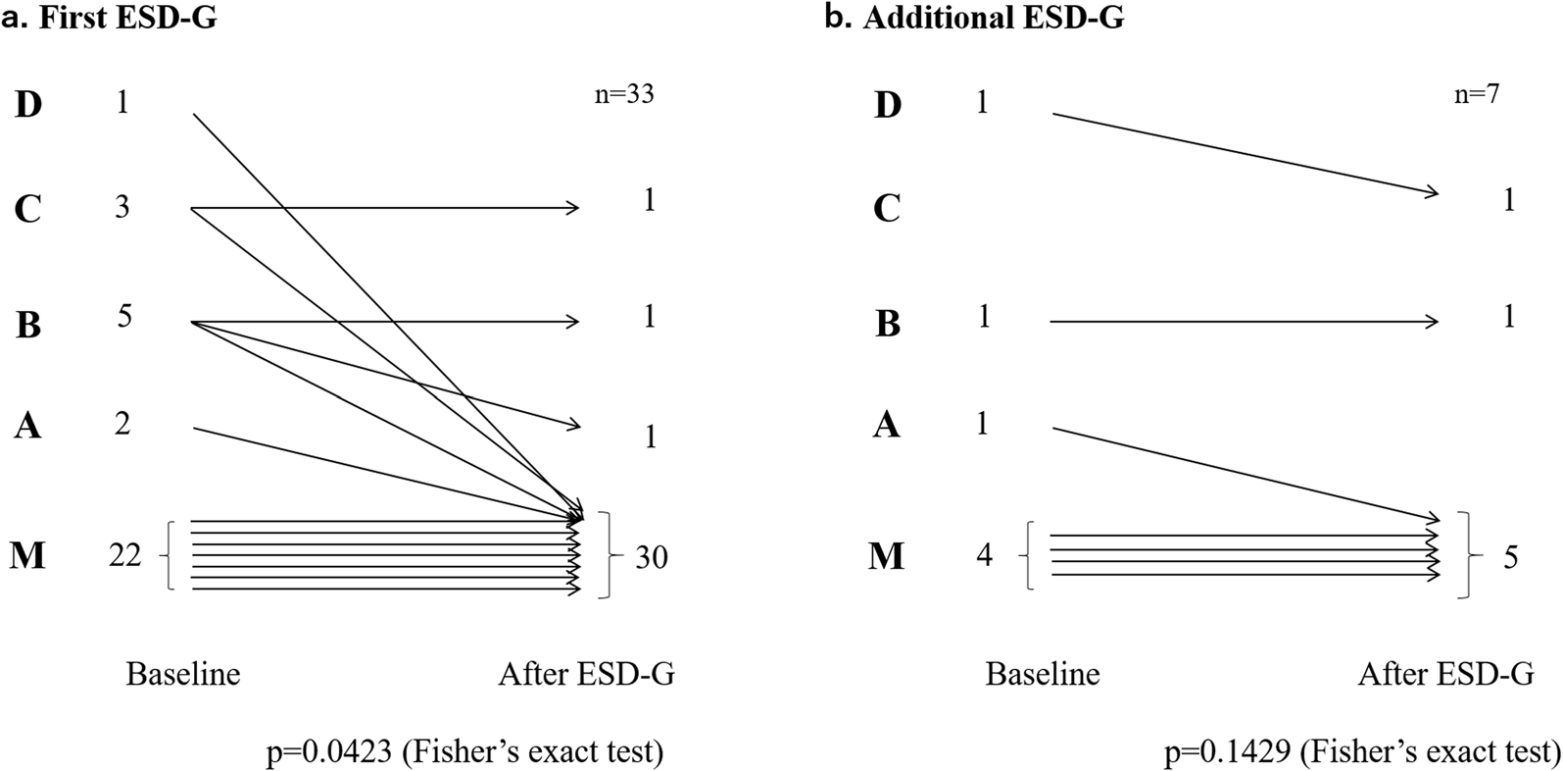
Los Angeles classification for reflux esophagitis is clearly improved after the first endoscopic submucosal dissection for gastroesophageal reflux disease (ESD-G) (*p* = 0.0423, Fisher exact test) (**a**). However, the effect of the additional ESD-G is likely to be poor (*p* = 0.1429, Fisher exact test) (**b**)

We analyzed 35 cases of first ESD-G. The patient characteristics were as follows: 25 patients (71.4%) were male, with a mean age of 51.5 (SD: 16.4) years, and mean body mass index was 21.8 (SD: 3.33) kg/m^2^ (Table 1). FSSG was assessed in detail before and after the procedure in 19 cases. The FSSG score was significantly improved 3–6 months after ESD-G. Surprisingly, not only the acid reflux score but also the dysmotility score was improved by ESD-G: acid reflux score, 15 [7–27] → 7 [0–23], *p* = 0.0012; dysmotility score, 10 [0–19] → 5 [0–16], *p* = 0.0013 (median [range], Wilcoxon signed-rank test). Endoscopic evaluation before and after the procedure was possible in 33 cases. The Los Angeles classification for reflux esophagitis was also significantly improved after ESD-G (*p* = 0.0423, Fisher exact test) (Fig. 2a). The 24-h esophageal MII monitoring study before and after the procedure was possible in 11 cases. The number of reflux episodes, both distal and proximal episodes, was not decreased by ESD-G: distal episodes, 54 [27–249] → 53 [34–252], *p* = 0.7793 and proximal episodes, 25 [4–97] → 27 [15–121], *p* = 0.6211 (median [range], Wilcoxon signed-rank test). In the comparison of the potency unit of gastric acid secretion suppressants for controlling GERD-related symptoms between before and 3–6 months after the procedure, there was a significant difference in the potency unit of gastric acid secretion suppressants for controlling GERD-related symptoms between baseline and 3–6 months after ESD-G in 34 patients: 2.73 ± 1.40 → 1.85 ± 1.61, *p* = 0.0009 (mean ± SD, paired *t* test). Seven patients were able to completely discontinue PPIs after ESD-G. We analyzed the only four patients who had a history of distal gastrectomy. Both the FSSG score and the potency unit of gastric acid secretion suppressants for controlling GERD-related symptoms did not improve after ESD-G (Table 2).

**Table 1:** Characteristics of patients included in this study

Number of cases	35
Male	25 (71.4%)
Age (SD)	51.5 (16.4)
Body mass index (SD)	21.8 (3.33)

*SD* standard deviation

**Table 2:** Other short-term outcomes of the first endoscopic submucosal dissection for gastroesophageal reflux disease

	Baseline (before ESD-G)	3–6 months after ESD-G	*p* value
Frequency scale for the symptoms of GERD (range)			
Acid reflux score, n = 19	15 (7–27)	7 (0–23)	0.0012^a^
Dysmotility score, n = 19	10 (0–19)	5 (0–16)	0.0013^a^
Potency unit of gastric acid secretion suppressants for controlling GERD-related symptoms (standard deviation)	2.73 (1.40)	1.85 (1.61)	0.0009^b^

*ESD-G* endoscopic submucosal dissection for gastroesophageal reflux disease

^a^Wilcoxon signed rank test

^b^Paired *t* test

Fifteen patients were followed after ESD-G for more than 5 years. Although there was a significant difference between before and 3–6 months after ESD-G in the potency unit of gastric acid secretion suppressants for controlling GERD-related symptoms in these patients (before ESD-G: 2.20 ± 1.37, 3–6 months after ESD-G: 1.43 ± 1.21, *p* = 0.0159; mean ± SD, paired *t* test), there was no significant difference before and more than 5 years after ESD-G (more than 5 years after ESD-G: 1.40 ± 1.76, *p* = 0.1828; mean ± SD, paired *t* test). In the only 12 patients with no history of distal gastrectomy, the potency unit of gastric acid secretion suppressants for controlling GERD-related symptoms was significantly decreased over time (before ESD-G: 2.25 ± 1.36, 3–6 months after ESD-G: 1.34 ± 1.03, more than 5 years after ESD-G: 0.75 ± 0.75; between before and 3–6 months after ESD-G, *p* = 0.0251; between before and more than 5 years after ESD-G, *p* = 0.0121, paired *t* test).

### Additional endoscopic submucosal dissection for gastroesophageal reflux disease

In seven patients, ESD-G was performed twice for the following various reasons: four patients had some improvement in GERD symptoms after the first ESD-G and requested an additional ESD-G procedure; two patients requested to undergo an additional ESD-G procedure because even though some of their GERD symptoms improved several years after the first ESD-G, their GERD symptoms were gradually worsening; and one patient requested to undergo additional ESD-G because the first ESD-G was not effective for his GERD symptoms (Table 3). Four of the seven patients had a history of distal gastrectomy. FSSG was assessed in detail before and after the procedure in four cases. There was no significant difference between before and 3–6 months after ESD-G (21.5 [21–28] → 13 [7–34], *p* = 0.3750, median [range], Wilcoxon signed-rank test). In the potency unit of gastric acid secretion suppressants for controlling GERD-related symptoms, there was also no significant difference between before and 3–6 months after ESD-G in seven patients (3.29 ± 1.26 → 3.57 ± 1.13, *p* = 0.3559, paired *t* test). There was no significant difference in the Los Angeles classification for reflux esophagitis between before and 3–6 months after ESD-G (*p* = 0.1429, Fisher exact test) (Fig. 2b). No patient was able to discontinue PPIs after additional ESD-G.

**Table 3:** Periods from the first ESD-G to the additional ESD-G and reasons for undergoing additional ESD-G

Case number of the patients who underwent ESD-G	Period from the first ESD-G to the additional ESD-G (days)	Reason for undergoing additional ESD-G	Reconstruction method after distal gastrectomy
1	161	Some improvement in GERD symptoms after the first ESD-G so patient requested additional ESD-G	Not available
2	176	Some improvement in GERD symptoms after the first ESD-G so patient requested additional ESD-G	Billroth 1
3	264	Some improvement in GERD symptoms after the first ESD-G so patient requested additional ESD-G	Billroth 1
4	399	Some improvement in GERD symptoms after the first ESD-G so patient requested additional ESD-G	Not available
5	763	Patient requested to undergo additional ESD-G because the first ESD-G was not effective for his GERD symptoms	Not available
6	2408	Patient requested to undergo additional ESD-G because some GERD symptoms improved several years after the first ESD-G but the GERD symptoms were gradually worsening	Billroth 1
7	3559	Patient requested to underwent additional ESD-G because some GERD symptoms improved several years after the first ESD-G but the GERD symptoms were gradually worsening	Billroth 1

*GERD* gastroesophageal reflux disease, *ESD-G* endoscopic submucosal dissection for gastroesophageal reflux disease

## Discussion

The present study revealed that ESD-G may be effective for patients with refractory GERD-related symptoms without a history of distal gastrectomy. To the best of our knowledge, this is the first study to investigate the clinical features of medication-refractory GERD patients for whom endoscopic treatment is effective in the long-term observational period.

We revealed that there was a significant improvement in GERD-related symptoms after ESD-G. However, this subjective outcome might have included some placebo effects. We used the potency unit of gastric acid secretion suppressants for controlling GERD-related symptoms as an objective indicator. There was also a significant reduction in the dosage of each PPI or vonoprazan after ESD-G. However, there was no improvement in the number of reflux episodes after ESD-G. These outcomes may mean that ESD-G did not decrease the number of refluxes, but rather reduced the GERD-related symptoms. The following are predicted to contribute to the improvement in GERD-related symptoms: (1) reduction of hypersensitivity in the lower esophageal mucosa caused by degeneration of the afferent nerve caused by ESD procedure, (2) reduction of volume of reflux content caused by a narrowing EGJ, and (3) dislocation of the acid reflux pathway from the hypersensitive mucosa due to scarring deformation of the lower esophagus. A similar discussion was reported previously [17]. Although there was no significant reduction in the number of gastroesophageal episodes according to the 24-h esophageal MII monitoring study, it is possible that the volume of reflux content per one reflux episode did decrease. As a further discussion, the sensitivity of gastric acid suppressant may be improved because not only the acid reflux score but also the dysmotility score was improved by ESD-G. To prove these, consideration of another gastrointestinal function examination is necessary. In patients who had discontinued PPIs for a long time after ESD-G without GERD symptoms, we considered that it might not be placebo effect by ESD-G because it was considered that GERD symptoms would eventually flare up in natural course if it was placebo effect in those patients.

The present study revealed that ESD-G may be less effective for patients with a history of distal gastrectomy than those without a history of gastric surgery. The remnant stomach is pulled downward by anastomosis with the intestinal tract and may reduce the narrowing of the EGJ lumen to scarring deformity by ESD-G. In addition, the remnant stomach lacks the pyloric ring, which may cause reflux of alkaline duodenal fluid to reach the esophagus via the remnant stomach. Different pathways may be involved in hypersensitivity to gastric acid and duodenal fluid, and ESD-G may only improve the former. GERD in the remnant stomach may require a different approach. For postoperative refractory reflux esophagitis with high duodenal reflux, the Roux-en-Y method of reconstruction should be considered [29, 30]. The anti-reflux mucosectomy (ARMS) reported by Sumi and Inoue et al. is a technique in which gastric mucosal resection is performed around the cardia to reshape its flap valve, which might make it more effective in suppressing reflux than our ESD-G [20, 21]. Because of these facts, in patients with a history of distal gastrectomy, it might be better to choose the ARMS procedure than the ESD-G if a patient insists on endoscopic treatment. In this study, the inclusion criteria of ESD-G was patients with endoscopic findings that is suggestive of the presence of GERD such as reflux esophagitis, cloudiness of the lower esophageal mucosa, or presence of Barrett’s epithelium and with GERD-related symptoms that persisted despite PPI therapy for 8 weeks. The PPI-refractory GERD might include functional heartburn and reflux hypersensitivity under this criteria. If ESD-G strengthens lower esophageal sphincter, it may also improve accommodation; therefore, ESD-G could possibly help improve functional heartburn [31]. As more cases are accumulated, the characters of patients who are refractory to ESD-G may become evident, as well as more stringent indications in the future.

A few patients developed complications from ESD-G. The ESD-G technique is highly challenging to perform; thus, less complicated and safer techniques need to be developed. The present study had some limitations. First, the sample size was small, as this was a single-center, single-arm study without a comparison group. Although we did not set up a control group, it might be possible that sham treatment could be set up in the following method, wherein endoscopy is performed without resection. Second, there are many missing data points, indicating that evaluation of some results is difficult. For example, impedance-pH monitoring was performed in < 50% of the enrolled patients, and the long-term effects of ESD-G, which is the primary outcome of this study, could be evaluated in only < 50% of the enrolled patients. Second, the mechanism by which ESD-G improves GERD-related symptoms remains ambiguous. In our study protocol, the diagnosis results, such as reflux esophagitis, true NERD, reflux hypersensitivity or functional heartburn, were not included in the analysis. Fourth, the results of the 24-h pH monitoring study could not be assessed because some patients were unable to withdraw from the gastric acid suppressants. We evaluated the efficacy of ESD-G by symptoms and PPI dose, not by endoscopy. In some cases, 24-h MII monitoring study was performed for the evaluation of ESD-G. In several cases, PPI could not be stopped preoperatively and pH monitoring was not available to evaluate the results in this study. However, for the assessment of GERD, impedance monitoring is equivalent to pH monitoring [32]. In addition, pH monitoring is not suitable for patients on PPI medication [33]. Therefore, we were able to analyze the results of the 24-h MII monitoring study, and we consider that the gastroesophageal reflux evaluation is adequate.

## Conclusion

ESD-G may be effective in patients with refractory GERD-related symptoms without a history of distal gastrectomy in the long term. Patients who undergo distal gastrectomy will have direct reflux of duodenal fluid, so gastroesophageal reflux needs to be more reliably controlled.

## Data Availability

The datasets used and analyzed during the current study will be available from the corresponding author on reasonable request.

## Abbreviations

PPI: Proton pump inhibitor
GERD: Gastroesophageal reflux disease
ESD-G: Endoscopic submucosal dissection for GERD
NERD: Non-erosive reflux disease
EGJ: Esophagogastric junction
FSSG: Frequency scale for the symptoms of GERD
MII: Multiple intraluminal impedance
SD: Standard deviation
ARMS: Anti-reflux mucosectomy
